# Evidence Accumulation in the Magnitude System

**DOI:** 10.1371/journal.pone.0082122

**Published:** 2013-12-05

**Authors:** Anna Lambrechts, Vincent Walsh, Virginie van Wassenhove

**Affiliations:** 1 Department of Psychology, City University London, London, United Kingdom; 2 Institute of Cognitive Neuroscience, University College London, London, United Kingdom; 3 INSERM U992, Cognitive Neuroimaging Unit, Gif/Yvette, France; 4 CEA, DSV/I2BM, NeuroSpin Center, Gif/Yvette, France; 5 Université Paris-Sud, Gif/Yvette, France; New York University, United States of America

## Abstract

Perceptual interferences in the estimation of quantities (time, space and numbers) have been interpreted as evidence for a common magnitude system. However, if duration estimation has appears sensitive to spatial and numerical interferences, space and number estimation tend to be resilient to temporal manipulations. These observations question the relative contribution of each quantity in the elaboration of a representation in a common mental metric. Here, we elaborated a task in which perceptual evidence accumulated over time for all tested quantities (space, time and number) in order to match the natural requirement for building a duration percept. For this, we used a bisection task. Experimental trials consisted of dynamic dots of different sizes appearing progressively on the screen. Participants were asked to judge the duration, the cumulative surface or the number of dots in the display while the two non-target dimensions varied independently. In a *prospective* experiment, participants were informed *before* the trial which dimension was the target; in a *retrospective* experiment, participants had to attend to all dimensions and were informed only *after* a given trial which dimension was the target. Surprisingly, we found that duration was resilient to spatial and numerical interferences whereas space and number estimation were affected by time. Specifically, and counter-intuitively, results revealed that longer durations lead to smaller number and space estimates whether participants knew before (prospectively) or after (retrospectively) a given trial which quantity they had to estimate. Altogether, our results support a magnitude system in which perceptual evidence for time, space and numbers integrate following Bayesian cue-combination rules.

## Introduction

Time, space, and numbers can be encoded through all sensory modalities. As such, these dimensions provide a first level of abstract quantification in mental space. Specifically, mental magnitudes can be defined as the neural realization of quantities which afford computational operations akin to arithmetic [1]–[4]. In recent years, several authors have postulated the existence of a common neural processing and representational scheme for mental magnitudes [2], [3], [5]–[7]. Among the dominant proposals, a Theory of Magnitude (ATOM) [7]–[8] argues that analog quantities are projected onto a common metric during development: through action, time and space are mapped onto a common pre-linguistic mental magnitude system and numerical processing maps out on an analogue continuum by capitalizing on the available magnitude system. ATOM predicts that magnitudes interfere with and prime one another. Alternatively, the Metaphor Theory (MT, [9]–[10]) proposes that a common magnitude mapping resides in the linguistic system: for instance, many languages use concrete spatial metaphors to express abstract temporal and numerical information [11]. MT thus predicts asymmetrical interferences between magnitude representations: space should dominate and strongly interfere with the temporal and numerical dimensions [12]. By far, the direction and the strength of interactions across dimensions remain unsettled and whether all quantities weigh equally in a common magnitude representational system is controversial. ATOM predicts comparable but not necessarily symmetrical interactions across magnitudes whereas MT specifically predicts asymmetries and yet others predict symmetrical interactions [13].

Space (size of stimulus, length of a line or a word, [11], [14], [15]) but also number (number of items, Arabic figure, [15]–[19]) have been shown to affect the estimation of duration: the larger the size of a stimulus or the number of items, the longer the perceived duration. Similarly, space and numbers interfere with each other such that the larger the size of a stimulus, the larger the perceived numerosity and reciprocally [20], [21]. In contrast, only one study [22] (recently extended [23]) has reported duration interference with numerical judgment: time appears to be the least reliable dimension i.e. the most susceptible to interference and the least influential on other magnitudes.

Here, we wanted to test whether duration could affect space and number estimations. First, we departed from the observation that in building an internal representation of duration, evidence accumulation through time was obligatory. In a majority of studies however, this property was neither addressed nor equated across magnitudes. In particular, spatial and numerical information have mostly been displayed as a single snapshot of varying duration (but see [11]). While all information for space and number estimation was available in the shortest amount of time (i.e. the time necessary to reach the internal criterion for reliable classification), perceptual evidence for duration necessarily had to go through an accumulation process. Hence, we insured that evidence accumulation was necessary for all magnitudes. For this, we designed stimuli consisting of a dynamic population of dots. This population was characterized by its duration, its cumulative surface (space) and the total number of dots composing it. In contrast to other studies, all three magnitudes were experimentally manipulated simultaneously (i.e. within a single trial) in order to investigate the combined influence of two types of magnitudes (e.g. space and time) on a third target magnitude (e.g. number). Crucially, task difficulty was equated across all three magnitudes by individually calibrating the discriminability of each magnitude stimuli (Weber Ratio, see Methods).

Two experiments were conducted to investigate the influence of cognitive load on this task. In effect, current models of time perception predict that diverting attention away from temporal estimation should affect the perception of duration: specifically, the more events within a time interval (i.e. the higher the cognitive load), the longer the estimated duration irrespective of the nature of these events [24]. To investigate whether cognitive load was particularly deleterious in duration estimation, two groups of participants were tested in a prospective and a retrospective variation of the main task. In the *prospective* experiment, participants were told *before* each trial which magnitude had to be estimated. Conversely, in the *retrospective* experiment, participants were informed *after* the trial which magnitude had to be estimated. Note that we use *retrospective* in a non-classical sense, namely as a factor affecting cognitive load and not as the absolute uncertainty about the stimulus feature to be estimated [25], [26]. Hence, in the prospective experiment, participants could focus on one of the three dimensions at the beginning of a given trial and could ignore orthogonal dimensions (low cognitive load); to the contrary in the retrospective experiment, participants had to attend all three dimensions in a given trial (high cognitive load) and could only retrospectively select one of them to provide their answer after instruction.

## Methods

### Participants

33 participants were recruited from local universities and compensated for their time. Participants provided their written consent in accordance with the Declaration of Helsinki (2008) and the study was approved by the Ethics Committee on Human Research review boards at NeuroSpin (Gif-sur-Yvette, France) and UCL (London, UK). Each participant only took part in one of the two experiments. Each experiment consisted in two sessions which took place on different days within the same week. Taking both experiments together, 3 participants' data were excluded from the study due to poor performance after the first session, (criterion of Weber Ratio>1 in all three experimental blocks), and 1 participant's data were excluded because he did not complete the second session. Data from 2 participants in experiment 1, and 3 participants in experiment 2 were excluded due to poor performance in the second session (cf. Analysis for criterion). Thus, 24 participants were considered in the study (11 males, age  = 23.5±3.8, 12 participants in each experiment).

### Stimuli

The experiment was coded using Matlab 7.0 and Psychtoolbox 3.0 [27]–[29]. Visual stimuli consisted of a cloud of grey dots appearing dynamically on a black screen. One trial was characterized by its duration (time elapsed between the appearance of the first dot and the disappearance of the last one), surface (cumulative surface covered by all dots) and numerosity (total number of dots appearing during the duration of the trial). All properties were chosen pseudo-randomly for each trial. The relative luminance of dots on each trial took one of 6 possible values: 57, 64, 73, 85, 102 and 128 in the 0(black)-to-255(white) RGB-coded referential. Dots appeared within a virtual disk of radius 5.7 to 7.7 degree of visual angle, and no dots could appear within an invisible protective inner disk of 0.9 degrees maintained around the central fixation at all times. Hence, neither luminance nor spatial density correlated with the surface or number of dots. The position of the dots was constrained so that two dots could not overlap in space or time; each dot had a limited lifetime of 333 ms. Accumulation of evidence was made irregular by adding new dots progressively, 2 to 7 at a time, in 9 to 13 steps. The duration and radius of each dot was chosen non-uniformly between 40 ms to 267 ms and 0.45 to 2.84 degrees, respectively.

### Experimental Design

**Figure 1:**
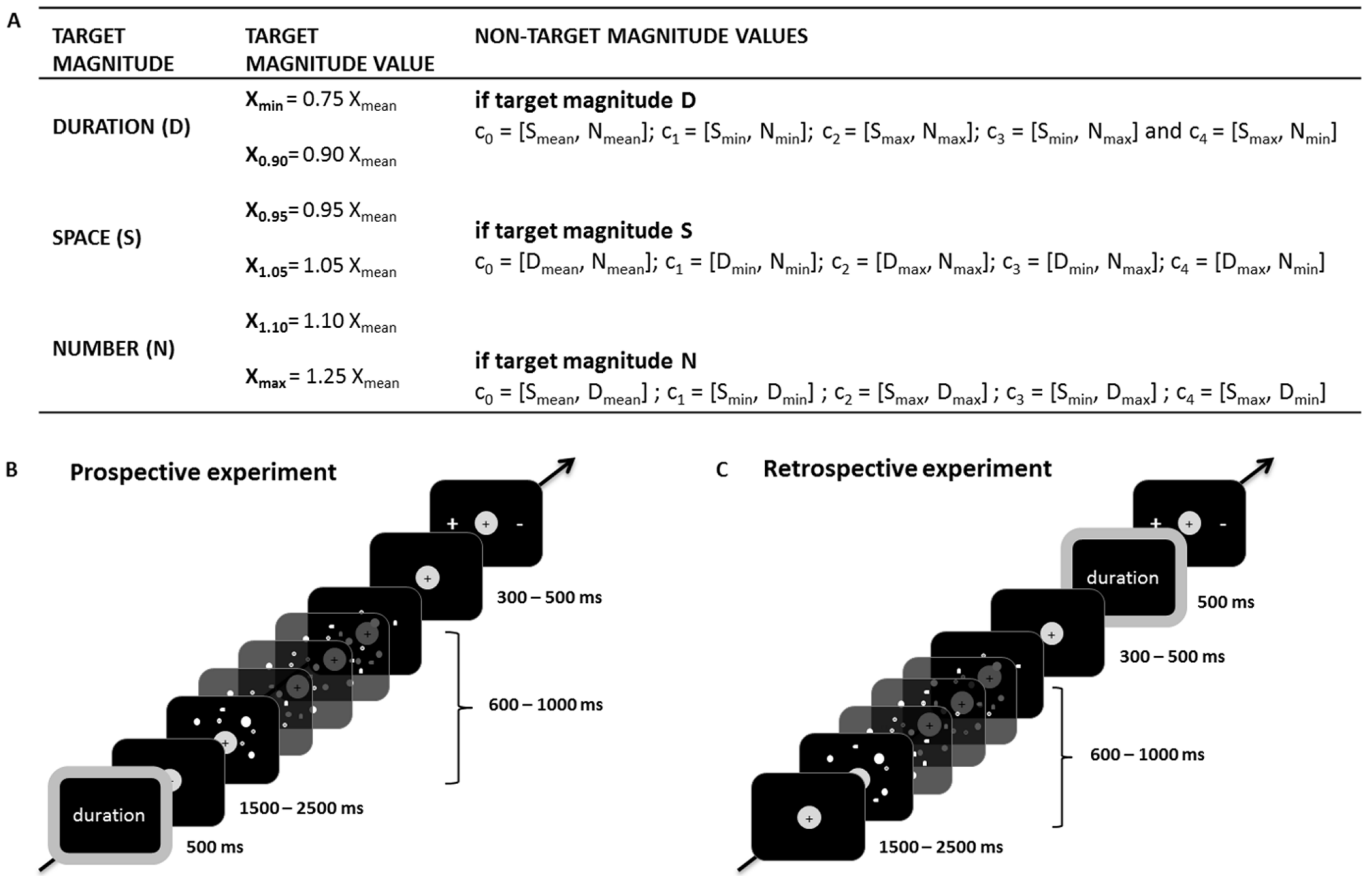
Experimental procedure. Panel A: the target magnitudes were either duration (D), space (S) or number (N). For each target magnitude six values were tested to draw reliable psychophysical thresholds: 0.75, 0.9, 0.95, 1.05, 1.1 and 1.25 times the target magnitude's mean value. Each of the six values was tested with five different possible combinations of the non-target magnitudes. Panel B: Prospective experiment. At the outset of a trial, participants were told which magnitude was to be estimated. Panel C: Retrospective experiment. Participants were told only after a given trial which magnitude needed to be estimated.

The paradigm was a bisection task (Figure 1). Each target dimension (duration (D), surface (S) and number (N)) took 6 possible values defined as 0.75, 0.9, 0.95, 1.05, 1.1 and 1.25 times the mean value (hereafter: X_0.75_, X_0.9_, X_0.95_, X_mean_, X_1.05_, X_1.1_ and X_1.25_, with dimension X being D, S or N; **Fig. 1a**). In the pre-test, participants were familiarized with the minimum (X_0.75_) and maximum (X_1.25_) values for each dimension (see Procedure section). In the subsequent tests, participants made a categorical judgment on one of the dimensions: ‘closer to the minimum (−)’ or ‘closer to the maximum (+)’ by pressing one of two keys. Magnitude estimation could be *prospective* (participants knew the target magnitude in advance; **Fig. 1b**) or *retrospective* (participants knew the target magnitude at the end of a trial; **Fig. 1c**). Five conditions were designed to explore the combined influence of irrelevant dimensions on the target magnitude judgment (**Fig. 1a**). In control *condition 0* (c_0_), orthogonal dimensions were set to their mean (Y_mean_, Z_mean_); in *condition 1* (c_1_), to their minimal values (0.75× mean value: Y_min_, Z_min_) and in *condition 2* (c_2_), to their maximal values (1.25× mean value: Y_max_, Z_max_). In *conditions 3 and 4* (c_3_, c_4_), one orthogonal magnitude value was maximal (Y_max_) whereas the other was minimal (Z_min_). The last two conditions allowed us to evaluate the relative weight of each orthogonal dimension.

### Procedure

Stimuli were displayed on a 1024×768 monitor screen with a 75 Hz frame rate. Participants were seated 60 cm away from the display. Response keys were ‘h’ and ‘j’ keys on the computer keyboard. Each experiment was carried out in two sessions taking place on different days within the same week.

In the first session, stimuli were adjusted individually using the measured participant's Weber Ratios (WR, see Analysis) to equate task difficulty for all three magnitudes. For this, participants were first familiarized with the minimum (−) and maximum (+) values in each dimensions (pre-test), based arbitrarily on T_mean_ = 800 ms, S_mean_ = 900 mm^2^ and N_mean_ = 28 dots. Three blocks of a short bisection task were then performed independently on each dimension X while orthogonal dimensions were held constant (set to Y_mean_ and Z_mean_). At the end of each block, for each participant, the WR was extracted and S_mean_ and N_mean_ were increased or decreased to calibrate task difficulty, resulting in identical WR all three dimensions. D_mean_ was kept constant (800 ms) for all participants. The final S_mean_ and N_mean_ were 878±105 mm^2^ and 27±3 dots respectively.

In the second session, participants performed a pre-test again to recalibrate the minimum and maximum values in each dimensions. They then performed a bisection task in which trials were pseudo-randomized across dimensions and conditions. A total of 900 trials were collected (3 magnitudes × 5 conditions × 6 values × 10 trials) in 100-trial blocks.

The instruction ‘Duration’, ‘Surface’ or ‘Number’ was displayed centrally on the monitor screen either before (Prospective experiment (**Fig. 1b**)) or after (Retrospective experiment (**Fig. 1c**)) a given trial. Participants were prompted for their response with the simultaneous appearance of ‘+’ and ‘−’ displayed on each side of the fixation cross. The relative position of ‘+’ and ‘−’ was pseudo-randomly assigned throughout the trials to avoid any bias due to congruency or incongruency between hand side and response. Participants were instructed at the beginning to avoid counting and to respond by hunch. There was no time constraint to respond. Reaction times (RT) were recorded.

### Analysis

Proportions of ‘+’ responses were computed separately per experiment, dimension and condition. Values were individually fitted to a logistic function *f* using Psignifit 3.0.8 [30] in Matlab 7.0. Two indices were computed: the Point of Subjective Equality (PSE, value at 50% of ‘+’ responses) and the Weber Ratio (WR). The WR was computed as half the distance between the values that support 25% and 75% of ‘+’ responses normalized by the PSE [31]–[33]. The closer WR is to 0, the greater the response accuracy.


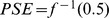
                   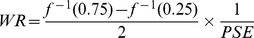


PSE, WR and RT data for which values were negative or outside ±3 standard deviations of the mean in each condition were replaced by the mean of the other values in the same condition. Participants for whom more than half the measures failed the criterion were excluded from the analysis. There was no more than one value replaced per condition.

Repeated-measure Analyses Of Variance (ANOVAs) were performed on PSEs, WRs and RTs using the IBM SPSS software (Version 19.0). A Greenhouse-Geisser correction was applied when appropriate. Post-hoc Bonferroni-corrected paired t-tests were performed to explore significant main effects or interactions.

## Results

**Figure 2:**
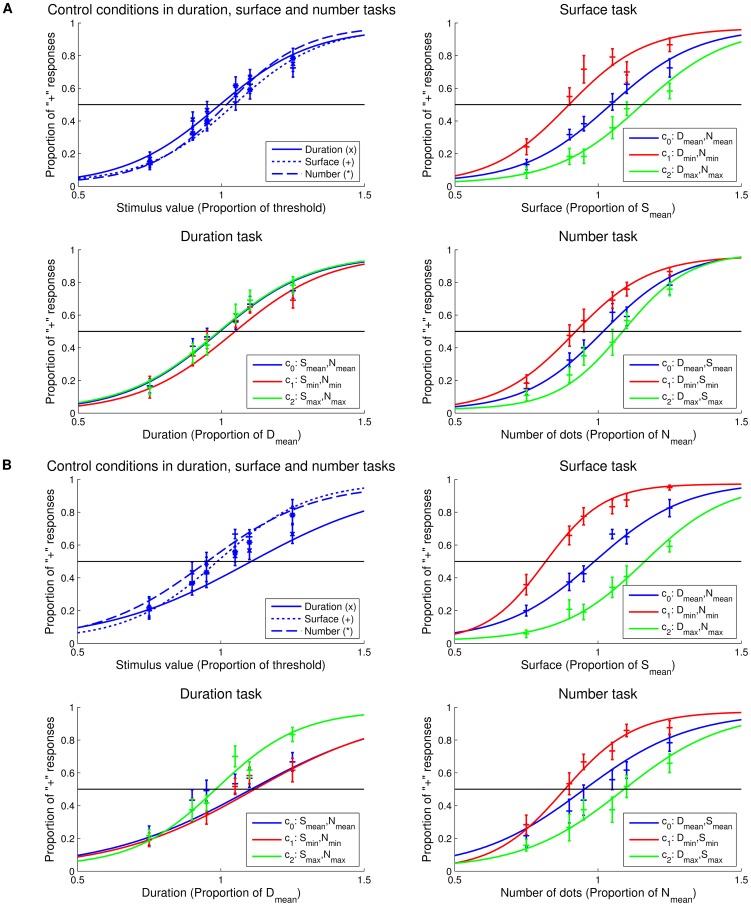
Mean psychometric curves. Psychometric curves for controls and conditions c_0_ (blue), c_1_ (red) and c_2_ (green) in the Duration (lower left), Surface (upper right) and Number (lower right) tasks. For display, sigmoid curves use the average fitting parameters across participants (n = 12). **Panel A**: Prospective Experiment. In the control conditions (upper left), no significant differences were observed when comparing all three magnitudes. **Panel B**: Retrospective experiment. In the control conditions (upper left), no significant differences were observed when comparing all three magnitudes. Bars are two s.e.m.

Repeated-measure ANOVAs with WR as dependent variable and factors of magnitude (3: D, S, and N) and condition (5) were conducted separately for the prospective and retrospective experiments. No main effects or interactions were found. This strongly suggests that participants’ sensitivity to the tested magnitudes did not vary across tasks and conditions, indicating that task difficulty was successfully matched across magnitudes (Fig. 2a and Fig. 2b).

### PSE analysis

#### Prospective magnitude estimation

In the prospective task, participants were instructed before each trial which magnitude they had to estimate. Repeated-measure ANOVA with PSE as dependent variable and factors of magnitude (3: D, S and N) and condition (5) revealed a main effect of condition (F_12,4_ = 5.327, p = .015, η_p_^2^ = .326) and a marginal interaction of magnitude with condition (F_12,8_ = 2.905, p = .051, η_p_^2^ = .209). Overall, manipulating orthogonal magnitudes significantly influenced the target magnitude estimation but this effect was not consistently observed for each target magnitude (Fig. 2a).

In **surface estimation**, PSE_0_ (D_mean_, N_mean_) were significantly higher than PSE_1_ (D_min_, N_min_) (t_12,11_, p<.001): surfaces were surprisingly overestimated when few dots were presented for a short duration. Additionally, surfaces were judged to be smaller when duration and number were maximal than when they were minimal (PSE_2_>PSE_1_, t_12,11_ = −3.389, p<0.01). Similarly, in **numerosity estimation**, PSE_0_ (D_mean_, S_mean_) were significantly higher than PSE_1_ (D_min_, S_min_) (t_12,11_ = 5.814, p<.001): numerosity was overestimated when the surface and the duration of dots were smallest. PSE_1_ (D_min_, S_min_) were also significantly lower than PSE_2_ (D_max_, S_max_) (t_12,11_ = −5.559, p<.001), suggesting that numerosity was estimated to be largest when duration and surface were smallest. PSE_1_ (D_min_, S_min_) were significantly smaller than PSE_4_ (D_max_, S_min_) (t_12,11_ = −8.218, p<.001), showing that with longer durations, numerosity was underestimated. Unexpectedly, neither surface nor numerosity significantly interfered with **duration estimation**. Hence, two surprising observations were that duration estimation appeared resilient to changes in other dimensions (see also Fig. S2) whereas surface and numerosity were both affected by changes in other dimensions.

In a second experiment, we asked whether this pattern of findings was solely based on prior expectation with regards to the magnitude to be estimated, or whether it held when the target dimension remained uncertain until after the stimulus had been displayed.

#### Retrospective magnitude estimation

In this task, participants were informed after a trial had passed which magnitude had to be estimated. As previously, repeated-measure ANOVA with PSE as dependent variable and factors of magnitude (3) and condition (5) were conducted. A main effect of condition (F_12,4_  = 7.721, p≤0.001, η_p_^2^ = .412) and a significant interaction of magnitude with condition (F_12,8_ = 8.683, p≤0.001, η_p_^2^ = .441) suggested that manipulating orthogonal dimensions significantly affected target magnitude estimation (Fig. 2b).

**Figure 3:**
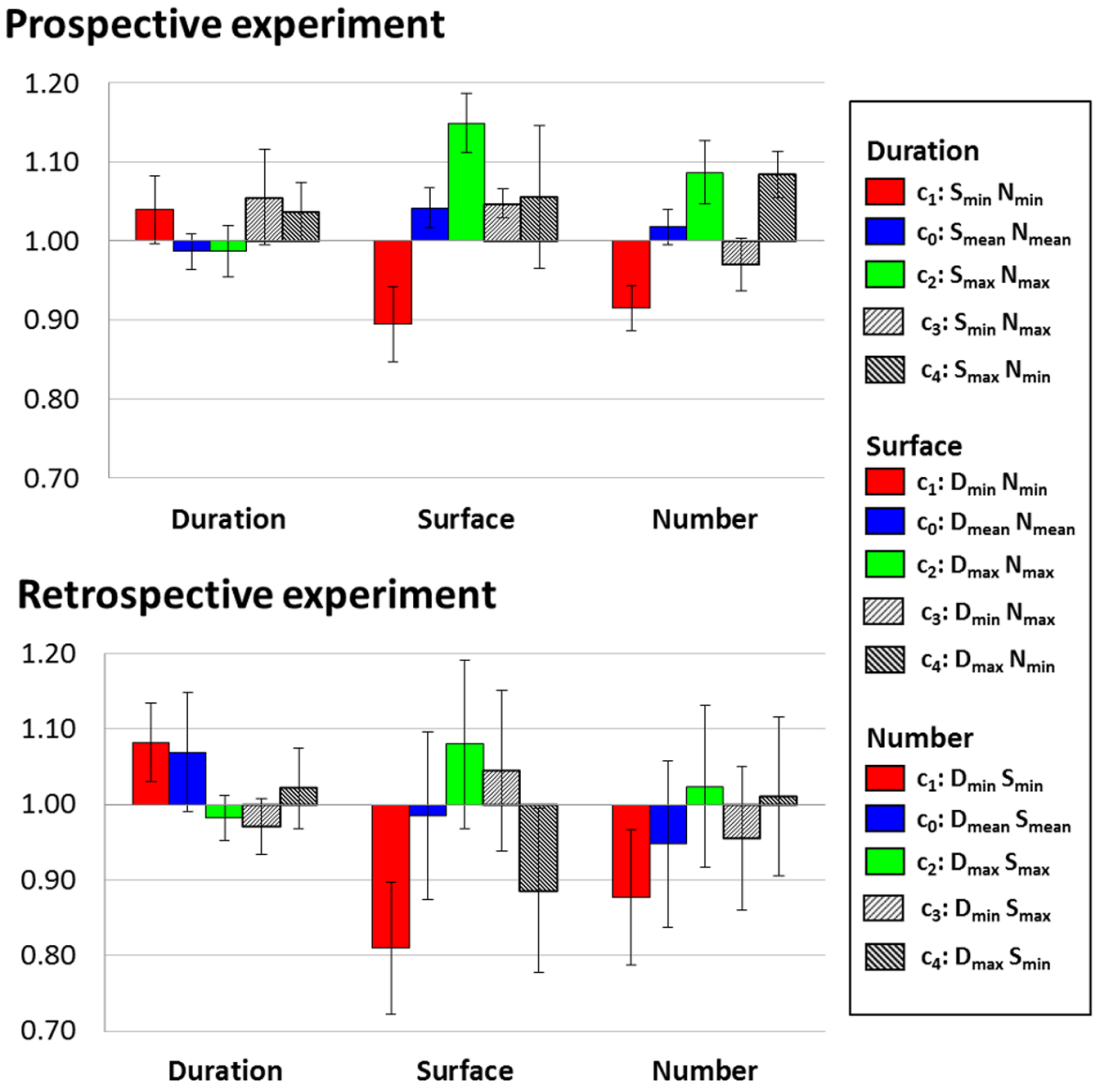
Point of Subjective Equality (PSE). PSE for conditions c_0_ (blue), c_1_ (red), c_2_ (green), c_3_ (right slanted stripes) and c_4_ (left slanted stripes) in the Duration (left), Surface (center) and Number (right) tasks. Note the gradation between c_1_, c_0_ and c_2_: the larger the Duration and Number of dots, the smaller the Surface estimate and the larger the Duration and Surface, the smaller the estimate for Number of dots. Bars are two s.e.m.

In **surface estimation**, all PSE significantly differed from one another (all p values ≤.005). As can be seen in Figure 3, PSE progressively increased from c_1_ (D_min_, N_min_), c_4_ (D_max_, N_min_), c_0_ (D_mean_, N_mean_), c_3_ (D_min_, N_max_) to c_2_ (D_max_, N_max_). Specifically, surfaces were overestimated when presented with few dots for a short duration but underestimated when presented with many dots for a long duration (PSE_1_<PSE_0_ and PSE_2_>PSE_0_ respectively). Consistent with the prospective experiment, combined duration and numerosity negatively interfered with surface estimation. Additionally, these results suggest that numerosity interfered more with surface estimation than duration did: when the number of dots was minimal (PSE_1_ and PSE_4_), surfaces were overestimated in comparison to PSE_0_ irrespective of duration; when the number of dots was maximal (PSE_2_ and PSE_3_), surfaces were underestimated relative to PSE_0_ irrespective of duration.

Similarly in **number estimation**, PSE_0_ (D_mean_, S_mean_) were lower than PSE_2_ (D_max_, S_max_) (t_12,11_ = −3.932, p<.005) and PSE_1_ (D_min_, S_min_) were lower than PSE_2_ (D_max_, S_max_) (t_12,11_ = −3.807, p<.005) and PSE_4_ (D_max_, S_min_) (t_12,11_ = −4.519, p<.005). In comparison to c_0_, numerosity was underestimated when surface was maximal over the longest duration (PSE_2_>PSE_0_); numerosity was smallest when surface and duration were largest (PSE_2_>PSE_1_). Duration had a stronger influence than surface on numerosity: a change in duration produced a significant change in estimates of numerosity (PSE_1_<PSE_4_) whereas a change in surface alone did not significantly interfere with numerosity. Overall, both surface and duration negatively interfered with numerosity estimations with a predominant effect of duration.

In **duration estimation**, PSE_1_ (S_min_, N_min_) were significantly higher than PSE_3_ (S_min_, N_max_) (t_12, 11_ = 3.922, p≤.005): for the smallest surface, duration estimation increased with number of dots. Thus numerosity influenced duration in the same direction (the more dots, the longer the duration). However, the absence of any other difference between conditions (in particular none involving conditions c_1_ and c_2_) indicates that orthogonal magnitudes interfered very little with duration estimation, in agreement with results in the prospective task.

Overall, the trends in PSE changes were comparable in both experiments: surface and numerosity showed little-to-no interference with the estimation of duration (see also Fig. S2) whereas duration and numerosity (Fig. S1A), and duration and surface (Fig. S1B), negatively interfered with estimation of surface and numerosity, respectively. Specifically, surface and numerosity were systematically over- or under-estimated when one or both non-target dimensions were smaller or larger, respectively. A summary of the effects is provided in Table S1.

Importantly, overall performance was not affected by task manipulation: a 2 (prospective vs. retrospective) × 3 (D, S, N) × 5 (conditions) mixed-design repeated-measure ANOVAs with PSE and WR as dependent variables revealed no main effect or interaction involving the factor experiment (prospective vs. retrospective). This negative finding suggests that increasing the cognitive load by attending to all three rather than one magnitude did not impact participants' performance or pattern of responses.

Below, we report the analysis of reaction times in both experiments (Fig. 3). RT measurements were initiated at the response prompt. In the retrospective experiment, participants had to maintain information on the three magnitude dimensions for ∼800–1000 ms (blank screen and instruction frame) before selecting the relevant information. In the prospective experiment, participants could focus on the relevant dimension beforehand. Therefore, RTs reflect different processes.

### RT analysis

#### Prospective magnitude estimation

**Figure 4:**
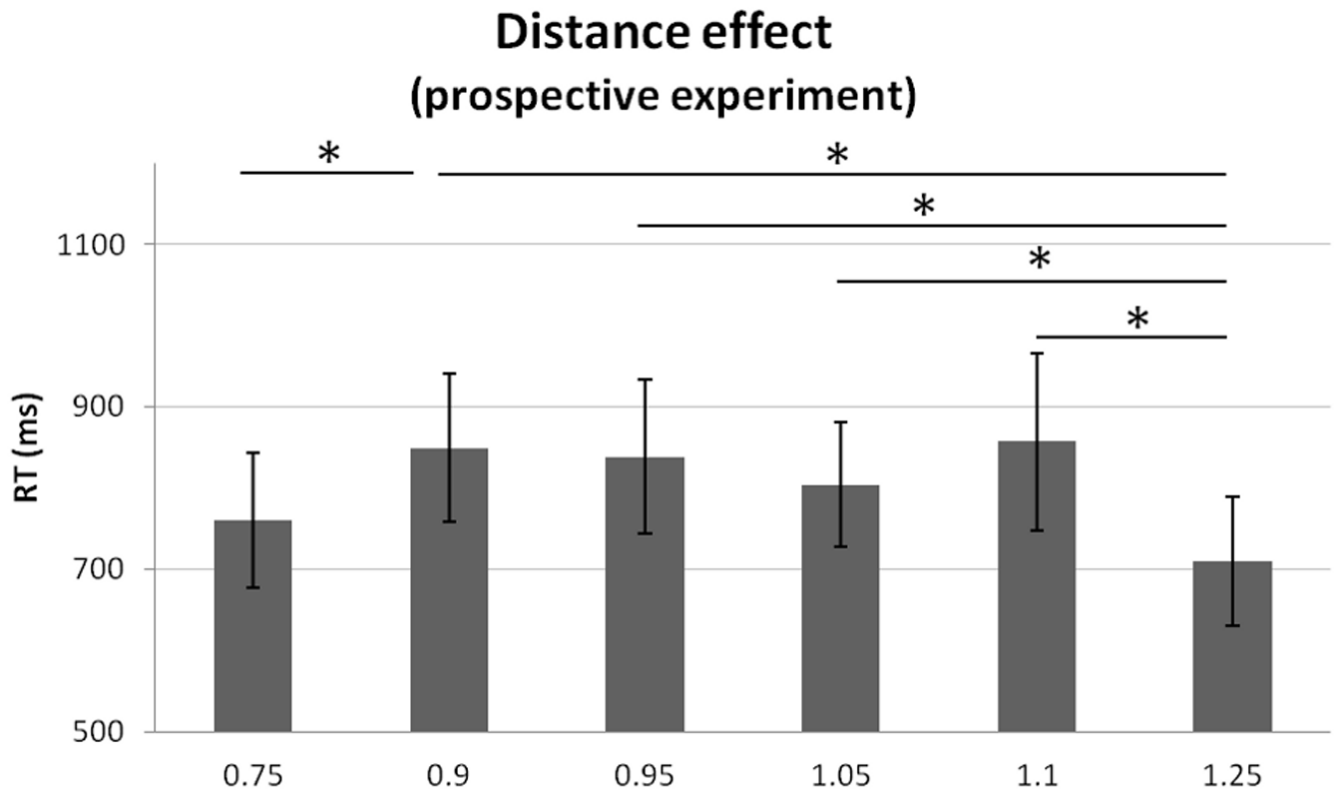
Distance effect in the prospective experiment. In all three Duration, Surface and Number prospective tasks, reaction times (RTs) were shorter when target stimuli were close to the anchor stimuli (0.75 and 1.75) than when the stimuli were in-between. Stars (*) indicate significant differences (p<0.05) as a result of Bonferroni-corrected t-tests. Bars are two s.e.m.

A repeated-measure ANOVA with RT as dependent variable and factors of magnitude (3: D, S, N) and magnitude quantity (6: 0.75, 0.9, 0.95, 1, 1.05, 1.1 and 1.25) revealed a main effect of quantity (F_12,5_ = 8.743, p≤.001, η_p_^2^ = .443). Paired Student t-tests across tasks showed that participants responded significantly faster to X_0.75_ than to X_0.9_ (t_12,11_ = −4.729, p≤.001), and that X_1.25_ was responded to significantly faster than X_0.9_, X_0.95_, X_1.05_ and X_1.1_ (t_12,11_ = 4.302, 5.960, 4.377 and 5.220 respectively, all p values ≤0.001). This is consistent with the distance effect [34], [35]: stimuli further from the discrimination threshold are easier to discriminate and elicit faster responses than stimuli closer to the threshold (Fig. 4).

#### Retrospective magnitude estimation

**Figure 5:**
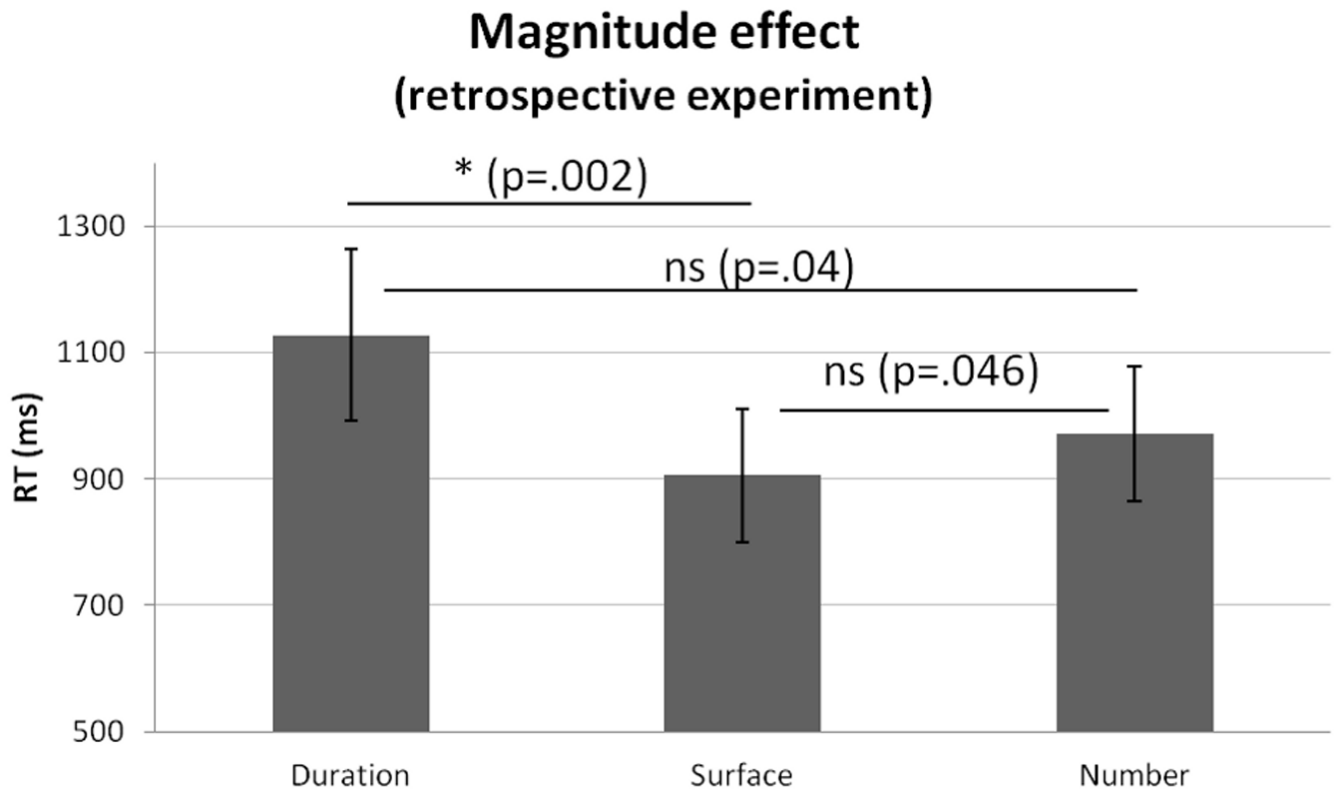
Magnitude effect in the retrospective experiment. When participants were informed of the target magnitude after the display, their RTs in the Duration task (left) were significantly longer than in the Surface task (center). Star (*) indicates a significant difference as a result of a Bonferroni-corrected t-test. Bars are two s.e.m.

A repeated-measure ANOVA with RT as dependent variable and factors of magnitude (3) and quantity (6) revealed a main effect of magnitude (F_12,2_ = 9.416, p≤.01, η_p_^2^ = .461) and of quantity (F_12,5_ = 2.584, p<.05, η_p_^2^ = .190). Paired Student t-tests across tasks showed that participants responded significantly faster to X_0.75_ than to X_0.9_ (t_12,11_ = −3.739, p≤.003). No other differences were observed indicating that RTs were little affected by quantity. However, paired Student t-tests across quantities showed that participants were significantly faster to estimate surface than duration (t_12,11_ = 4.108, p<.005) suggesting a magnitude effect in which duration is longest to retrieve when all three dimensions are held in memory (Fig. 5).

## Discussion

We investigated how time, space and numbers prospectively and retrospectively interacted with one another in a magnitude bisection task. Participants were asked to provide categorical judgments on a target dimension namely, duration, cumulative surface, or number while the other two dimensions were manipulated. Three main factors of interest were: equated difficulty across magnitude dimensions, forced evidence accumulation for all magnitudes and manipulation of cognitive load.

First, one main result for this study is that duration estimation was resilient to spatial and numerical information whereas surface and number estimations were sensitive to duration changes. These results are in stark contrast with previous findings in which time estimation was reported to be highly sensitive to concomitant spatial and numerical manipulations [11], [15]–[19]. In most studies, spatial and numerical information were immediately available whereas here, spatial and numerical information accumulated over time and were fully accessible only at the end of a given trial. With this manipulation, the time to reach perceptual decision was comparable for all three magnitudes and results show that under such constrains, space and number do not interfere with time estimation. One possible interpretation for these results is that the encoding of duration is independent from other magnitudes. For instance, in the retrospective experiment, RTs were larger for duration than for surface estimations. However, no such RT differences were observed in the prospective experiment, suggesting that the retrieval but not the encoding of duration differs from other dimensions. The difference in RTs in the retrospective experiment could indicate that the retrieval of temporal information may not be “prioritized” and it could be argued that time is critical for online prospective monitoring. In contrast, the primacy of spatial information would arguably be necessary for spatial navigation and immediate adaptation of gait and movement to the geography of our environment (ATOM, [7]).

An alternative interpretation is that when the availability of magnitude evidence is incremental, time is encoded more reliably than other magnitudes. To the best of our knowledge, only one study [11] attempted to equate evidence accumulation across dimensions: participants had to judge the length or duration of a growing line. However, length estimation could be computed on the spatial coordinates of the first and last pixel independently of the “quantity of space” traveled by the line. Here, spatial and numerical information had to be computed dynamically and results crucially suggest that when space and number accumulate over time, duration estimates can be resilient to interference from other magnitudes.

A second unexpected result of this study was the directionality of the effects. Specifically, duration negatively influenced magnitude estimates so that the longer the duration, the smaller the surface and the number were estimated. In recent reports on time-number interference [22], [23], longer durations increased the estimated number of items. One possible interpretation for these results would be that during the course of a trial, spatial and numerical information decay over time: assuming that surface and number accumulate uniformly over time, the longer the duration, the greater the informational loss and the more surface and number would be underestimated. Under constrained evidence accumulation, this interpretation favors a dominant effect of duration on space and number encoding and it should be noted that duration undergoes a similar loss [36]. However, this alternative also eradicates the need for a common representational system of magnitudes. We temper this interpretation below.

First, while providing a minimalist account of the effect on space and number estimates, it is unclear why both space and numbers would show a comparable decay rate if not encoded through the same channel. Second, RT for time should be systematically shorter as time would be favored as a direct parameter (memory) compared to space and numbers (informational content). Third, a leaky loss of information over time would predict that the distance effect for short durations should be more pronounced than for larger durations: we computed the distance effect on space and number separately for minimal and maximal duration trials and did not observe any significant differences as a function of duration (Fig. S3). However, the number of trials may be insufficient to robustly conclude on this point. Fourth, this interpretation would suggest that informational density is crucial in the estimation of magnitude and this would need to be further explored. Fifth, duration estimates should always be underestimated with regards to the total amount of evidence being accumulated by virtue of memory decay and this is not what we observe as the PSE for the control condition is not significantly above 1 in our data; similarly PSE do not significantly differ from 1 in the control conditions of surface and number. Finally, the current experiment cannot entirely rule out the possibility for that interpretation and a specific experiment should be designed in order to address the effect of surface and number with these stimuli for a larger set of constant time interval. As an alternative, we propose that magnitude judgments rely on the integration of magnitude information accumulated over time and thus, on stimulus sampling. In this view, magnitude estimates become sensitive to local temporal density: when duration increased (decreased), the number of dots within a given time interval decreased (increased) on average, leading both surface and number to be underestimated. In a majority of studies in which spatial and numerical information were presented at once (for instance, in symbolic form), the local spatiotemporal density could not be affected by the duration of the stimulus [15], [16], [18], [19]. This, we contend, could explain the lack of substantial evidence for time interference with other magnitudes in past reports.

Interestingly, the increased cognitive load introduced in the retrospective experiment did no significantly interfere with any of the magnitude estimations as compared to prospective judgments. These results were unusual in light of time perception research: previous results showed that during a prospective time estimation task, increasing the cognitive load or driving attention away from temporal monitoring systematically leads to time compression [37], [38] whether or not the distracting stimulus is task-relevant [39] whereas in a retrospective task, increasing the cognitive load classically leads to time dilation [24]. In our study, duration estimation did not differ according to the paradigm, which suggests that the attentional load was similar in both experiments i.e. that all three dimensions were encoded automatically. Additionally in the retrospective experiment, the classic distance effect was replicated [34], [35] for all magnitudes, namely: stimuli close to the anchors showed shorter RTs than stimuli remote from the anchors. Hence, both sets of results support an automatic magnitude mapping in mental space.

How then can we reconcile the lack of interference on time estimation with an automatic magnitude mapping? Recent computational advances have successfully addressed the problem of multiple cues combination using Bayesian principles [40], [41]. This successful approach has been extended across sensory modalities [42] and independently applied to spatial, numerical and size judgments [43]–[45] and more recently to temporal judgments [46]. Of particular interest here is the measure of mental distance between internal representations, which has been proposed to predict which of cue integration or cue dominance would be most likely to take place during combination [47].

Applying an analogous principle to the magnitude system, magnitude representation could be estimated based on the integration of all quantities estimates. The weight of information provided by each magnitude dimension could depend on two factors: the precision of the estimate in the corresponding dimension, and the mental distance between dimensions – determined by how strong the system believes that information provided by one magnitude dimension is related to another. The smaller the mental distance, the more integration (or interference) across dimensions should be observed whereas the larger the mental distance, the more dominant a dimension should become in the representation of magnitude. In our task, the weights of time, space and number were equated by design (same Weber Ratios) but the belief was skewed by time as surface and number strongly depended on the time over which the evidence accumulated. Hence, this paradigm enabled to strengthen the belief of the system that duration inversely predicted other dimensions (the longer the duration, the smaller the surface/number of dots). Here, it is unclear whether the pattern of results fit an interpretation as cue integration (i.e. cue integration increases with the belief that duration predicts other dimensions) or as an “all-or-none” time-dominant effect (i.e. integration occurs when the strength of belief reaches a certain threshold). Irrespective, our results are compatible with the observation that time is rarely observed to affect spatial and numerical judgments yet can, under certain conditions, dominate quantity estimations. By far, most studies have used paradigms in which space or number were *de facto* dominant considering that full evidence was provided as soon as a stimulus was displayed. As such, most interactions were dominated by either space or number but seldom by time. By introducing a task in which time naturally dominated, the opposite direction was observed. We thus suggest that our results converge with a Bayesian principle for dimension integration in a common magnitude representation. For instance, negative interference of number on surface judgment was observed between space and numbers in the retrospective experiment. In our design, for a given number of dots, larger surfaces contained on average bigger dots yielding to an underestimation of cumulative surface; conversely, for a given surface, a large number of smaller dots on average were displayed yielding to an overestimation of surface. This compensatory mechanism could be predicted when evidence from multiple dimensions has to be integrated over time. Additional research could further explore the directionality of space and number interactions when they accumulate over time, for example by maintaining the duration constant and manipulate spatial and numerical information independently.

By imposing evidence accumulation on all magnitude estimations, the present study showed that time can become resilient and in fact strongly interfere with space and numbers. It is here proposed that the encoding of dimensions rely on cue-combination mechanisms ultimately leading to an integrated magnitude representation [2], [3], [5]–[7], [9], [10], [48], [49]. In this view, a straightforward experimental prediction is that the time at which a symbolic magnitude is presented during evidence accumulation for time should interfere more or less strongly with magnitude estimates as well as predict the direction of these effects.

## Supporting Information

Figure S1 **Scatterplots illustrating the effect of duration on spatial and numerical judgments.** Data points show individual PSE in the surface and number tasks in a condition where duration is maximal against a condition in which duration is minimal while surface or number are held constant. (**A**) Influence of duration on surface judgments in the prospective (top) and retrospective (bottom) experiments. On the left panels, number is maintained at maximal value (N_max_ = 1.25×N_mean_) whereas duration is either minimal (c_3_: D_min_ = 0.75×D_mean_) or maximal (c_2_: D_max_ = 1.25×D_mean_). On the right panels, number is maintained at minimal value (N_min_ = 0.75×N_mean_) whereas duration is either minimal (c_1_: D_min_ = 0.75×D_mean_) or maximal (c_4_: D_max_ = 1.25×D_mean_). (**B**) Influence of duration on number judgments in the prospective (top) and retrospective (bottom) experiments. On the left panels, surface is maintained at maximal value (S_max_ = 1.25×S_mean_) whereas duration is either minimal (c_3_: D_min_ = 0.75×D_mean_) or maximal (c_2_: D_max_ = 1.25×D_mean_). On the right panels, surface is maintained at minimal value (S_min_ = 0.75×S_mean_) whereas duration is either minimal (c_1_: D_min_ = 0.75×D_mean_) or maximal (c_4_: D_max_ = 1.25×D_mean_).
(TIF)

Figure S2 **Scatterplots illustrating the absence of spatial and numerical effects on duration judgments.** Data points show individual PSE in the duration task in a condition where surface (resp. number) is maximal against a condition in which surface (resp. number) is minimal while number (resp. surface) is held constant. (**A**) Influence of surface on duration judgments in the prospective (top) and retrospective (bottom) experiments. On the left panels, number is maintained at maximal value (N_max_ = 1.25×N_mean_) whereas surface is either minimal (c_3_: S_min_ = 0.75×S_mean_) or maximal (c_2_: S_max_ = 1.25×S_mean_). On the right panels, number is maintained at minimal value (N_min_ = 0.75×N_mean_) whereas surface is either minimal (c_1_: S_min_ = 0.75×S_mean_) or maximal (c_4_: S_max_ = 1.25×S_mean_). (**B**) Influence of number on duration judgments in the prospective (top) and retrospective (bottom) experiments. On the left panels, surface is maintained at minimal value (S_min_ = 0.75×S_mean_) whereas number is either minimal (c_1_: N_min_ = 0.75×N_mean_) or maximal (c_3_: N_max_ = 1.25×N_mean_). On the right panels, surface is maintained at maximal value (S_max_ = 1.25×S_mean_) whereas number is either minimal (c_4_: N_min_ = 0.75×N_mean_) or maximal (c_2_: N_max_ = 1.25×N_mean_).
(TIF)

Figure S3 **Influence of trial duration on distance effect in the surface (left) and number (right) tasks.** Distance effects have been computed separately for trials in which duration is D_min_ and trials in which duration equal D_max_. No significant difference was found between duration conditions for either task. Error bars show standard error of the mean.
(TIF)

Table S1 **Post-hoc t-tests comparing PSE in different conditions within each modality, for the prospective (A) and retrospective (B) experiments.** Results are Bonferroni-corrected (significance threshold  = .005). Values in the table are t values (p values).
(DOCX)
