# Supplementary figures and images for: Evidence Accumulation in the Magnitude System
Source: PLoS One. 2013 Dec 5;8(12):e82122. doi: 10.1371/journal.pone.0082122 (PMC3855382; doi:10.1371/journal.pone.0082122)

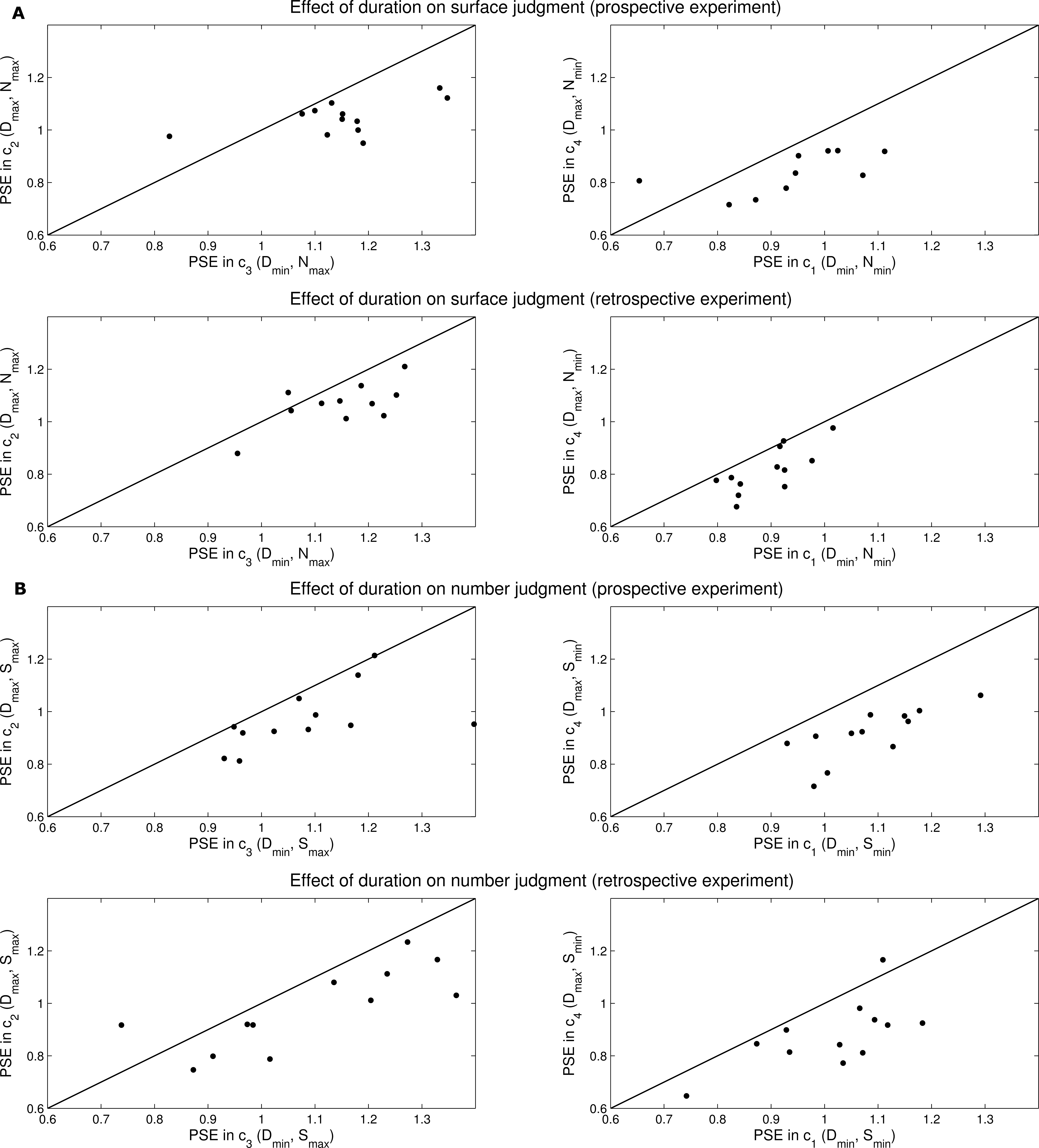

Supplement: Figure S1 — Scatterplots illustrating the effect of duration on spatial and numerical judgments. Data points show individual PSE in the surface and number tasks in a condition where duration is maximal against a condition in which duration is minimal while surface or number are held constant. (A) Influence of duration on surface judgments in the prospective (top) and retrospective (bottom) experiments. On the left panels, number is maintained at maximal value (Nmax = 1.25×Nmean) whereas duration is either minimal (c3: Dmin = 0.75×Dmean) or maximal (c2: Dmax = 1.25×Dmean). On the right panels, number is maintained at minimal value (Nmin = 0.75×Nmean) whereas duration is either minimal (c1: Dmin = 0.75×Dmean) or maximal (c4: Dmax = 1.25×Dmean). (B) Influence of duration on number judgments in the prospective (top) and retrospective (bottom) experiments. On the left panels, surface is maintained at maximal value (Smax = 1.25×Smean) whereas duration is either minimal (c3: Dmin = 0.75×Dmean) or maximal (c2: Dmax = 1.25×Dmean). On the right panels, surface is maintained at minimal value (Smin = 0.75×Smean) whereas duration is either minimal (c1: Dmin = 0.75×Dmean) or maximal (c4: Dmax = 1.25×Dmean). (TIF) [file pone.0082122.s001.tif]

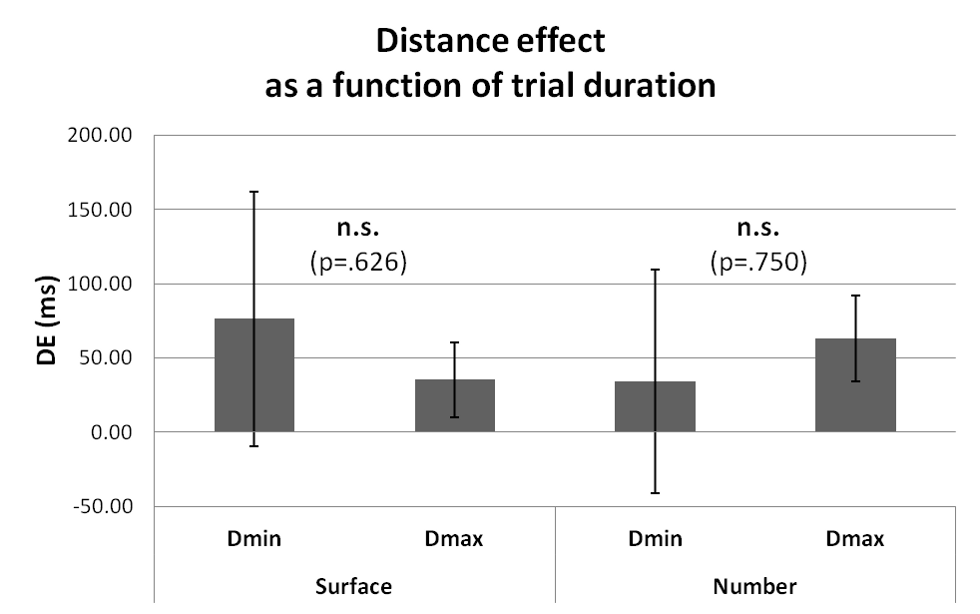

Supplement: Figure S3 — Influence of trial duration on distance effect in the surface (left) and number (right) tasks. Distance effects have been computed separately for trials in which duration is Dmin and trials in which duration equal Dmax. No significant difference was found between duration conditions for either task. Error bars show standard error of the mean. (TIF) [file pone.0082122.s003.tif]
