# Supplementary material for: Evidence Accumulation in the Magnitude System
Source: PLoS One. 2013 Dec 5;8(12):e82122. doi: 10.1371/journal.pone.0082122 (PMC3855382; doi:10.1371/journal.pone.0082122)
Supplement: Table S1 — Post-hoc t-tests comparing PSE in different conditions within each modality, for the prospective (A) and retrospective (B) experiments. Results are Bonferroni-corrected (significance threshold = .005). Values in the table are t values (p values). (DOCX) [file pone.0082122.s004.docx]

**A. Prospective experiment**

| ***Duration*** | **c_1_** (S_min_, N_min_) | **c_2_** (S_max_, N_max_) | **c_3_** (S_min_, N_max_) | **c_4_** (S_max_, N_min_) |
| --- | --- | --- | --- | --- |
| **c_0_** (S_mean_, N_mean_) | -1.570 (.145) | -0.032 (.975) | -1.105 (.293) | -1.704 (.116) |
| **c_1_** (S_min_, N_min_) |  | 1.936 (.079) | -0.344 (.737) | 0.047 (.964) |
| **c_2_** (S_max_, N_max_) | - |  | -1.292 (.223) | -1.269 (.231) |
| c**_3_** (S_min_, N_max_) | - | - |  | 0.219 (.830) |
| ***Surface*** | **c_1_** (D_min_, N_min_) | **c_2_** (D_max_, N_max_) | **c_3_** (D_min_, N_max_) | **c_4_** (D_max_, N_min_) |
| **c_0_** (D_mean_, N_mean_) | 5.550 (.000)* | -1.980 (.073) | -0.153 (.881) | -0.174 (.865) |
| **c_1_** (D_min_, N_min_) |  | -3.389 (.006) | -2.756 (.019) | -2.423 (.034) |
| **c_2_** (D_max_, N_max_) | - |  | 3.247 (.008) | 0.867 (.404) |
| c**_3_** (D_min_, N_max_) | - | - |  | -0.083 (.935) |
| ***Number*** | **c_1_** (D_min_, S_min_) | **c_2_** (D_max_, S_max_) | **c_3_** (D_min_, S_max_) | **c_4_** (D_max_, S_min_) |
| **c_0_** (D_mean_, S_mean_) | 5.814 (.000)* | -2.551 (.027) | 1.543 (.151) | -2.257 (.045) |
| **c_1_** (D_min_, S_min_) |  | -5.559 (.000)* | -1.419 (.184) | -8.218 (.000)* |
| **c_2_** (D_max_, S_max_) | - |  | 3.269 (.007) | 0.082 (.936) |
| c**_3_** (D_min_, S_max_) | - | - |  | -2.451 (.032) |

**B. Retrospective experiment**

| ***Duration*** | **c_1_** (S_min_, N_min_) | **c_2_** (S_max_, N_max_) | **c_3_** (S_min_, N_max_) | **c_4_** (S_max_, N_min_) |
| --- | --- | --- | --- | --- |
| **c_0_** (S_mean_, N_mean_) | -0.128 (.900) | 1.405 (.188) | 1.378 (.195) | 0.429 (.676) |
| **c_1_** (S_min_, N_min_) |  | 2.400 (.035) | 3.922 (.002)* | 0.947 (.364) |
| **c_2_** (S_max_, N_max_) | - |  | 0.279 (.785) | -1.514 (.158) |
| c**_3_** (S_min_, N_max_) | - | - |  | -1.876 (.087) |
| ***Surface*** | **c_1_** (D_min_, N_min_) | **c_2_** (D_max_, N_max_) | **c_3_** (D_min_, N_max_) | **c_4_** (D_max_, N_min_) |
| **c_0_** (D_mean_, N_mean_) | 9.713 (.000)* | -7.866 (.000)* | -3.516 (.005)* | 4.669 (.001)* |
| **c_1_** (D_min_, N_min_) |  | -21.825 (.000)* | -9.757 (.000)* | -4.694 (.001)* |
| **c_2_** (D_max_, N_max_) | - |  | 3.830 (.003)* | 15.874 (.000)* |
| c**_3_** (D_min_, N_max_) | - | - |  | 6.630 (.000)* |
| ***Number*** | **c_1_** (D_min_, S_min_) | **c_2_** (D_max_, S_max_) | **c_3_** (D_min_, S_max_) | **c_4_** (D_max_, S_min_) |
| **c_0_** (D_mean_, S_mean_) | 1.077 (.305) | -3.932 (.002)* | -0.682 (.509) | -1.369 (.198) |
| **c_1_** (D_min_, S_min_) |  | -3.807 (.003)* | -1.874 (.088) | -4.519 (.001)* |
| **c_2_** (D_max_, S_max_) | - |  | 3.040 (.011) | 1.697 (.118) |
| c**_3_** (D_min_, S_max_) | - | - |  | -0.865 (.405) |

Supplementary table 1

Results of post-hoc t-tests comparing PSE in different conditions within each modality, for the prospective (A) and retrospective (B) experiments. Results are Bonferroni-corrected (significance threshold = .005). Values in the table are t values (p values).
